# Management of Emotional Dysregulation in Adult ADHD

**DOI:** 10.1192/j.eurpsy.2022.1858

**Published:** 2022-09-01

**Authors:** F. Tavares, M. Viseu, M. Barbosa Pinto, C. Solana

**Affiliations:** 1 University Hospital Center of Algarve, Portugal, Department Of Psychiatry And Mental Health – Faro, Faro, Portugal; 2 University Hospital Center of Algarve, Portugal, Department Of Psychiatry And Mental Health - Faro, Faro, Portugal

**Keywords:** management, emotional dysregulation, Pharmacotherapy, attention-deficit/hyperactivity disorder

## Abstract

**Introduction:**

Attention-Deficit/Hyperactivity Disorder (ADHD) is characterized by impairing symptoms of inattention and/or hyperactivity/impulsivity. Although Emotional Dysregulation (ED) is not current criteria for ADHD, several clinical, imaging and genetic studies have been suggesting its inclusion. ED seems to impair social and occupational capacities, leading to poor quality life. In this regard, managing this situation is fundamental.

**Objectives:**

ED in ADHD review and its management, including pharmacological and nonpharmacological approaches.

**Methods:**

Non-systematic review through literature using databases as Pubmed and UpToDate. Keywords used: Attention-Deficit/Hyperactivity Disorder, Emotional Dysregulation, management, pharmacotherapy.

**Results:**

Literature refers to ADHD drugs, such as psychostimulants and atomoxetine, as the first line managing ED. However, some studies demonstrated that ADHD drugs have lower efficacy while treating emotional symptoms, when compared to attention or hyperactivity/impulsivity symptom control. Other medications, such as antidepressants or mood stabilizers, are not considered due to low efficacy and side effects (such as irritability or suicidality behaviour worsening). Regarding non pharmacological approaches, there have been results with cognitive behavioral treatment, and management techniques for anger, frustration and communication skills.

**Conclusions:**

Although the majority of studies demonstrate psychostimulants and atomoxetine role, there is an important lack of information regarding management of ADHD emotional dysregulation. It is a multifactorial condition, and, as such, non pharmacological and pharmacological management are needed to address this issue. More research is necessary, in order to improve patients’ quality of life.

**Disclosure:**

No significant relationships.

